# Oxalate as a potent promoter of kidney stone formation

**DOI:** 10.3389/fmed.2023.1159616

**Published:** 2023-06-05

**Authors:** Tao Chen, Biao Qian, Junrong Zou, Peiyue Luo, Jun Zou, Wei Li, Qi Chen, Liying Zheng

**Affiliations:** ^1^The First Clinical College, Gannan Medical University, Ganzhou, Jiangxi, China; ^2^Department of Urology, The First Affiliated Hospital of Gannan Medical University, Ganzhou, Jiangxi, China; ^3^Key Laboratory of Urology and Andrology of Ganzhou, Ganzhou, Jiangxi, China; ^4^Department of Graduate, The First Affiliated Hospital of Ganna Medical University, Ganzhou, Jiangxi, China

**Keywords:** oxalate, kidney stones, gut microbiome, SLC26A6, kindey, hyperoxaluria

## Abstract

Kidney stones are among the most prevalent urological diseases, with a high incidence and recurrence rate. Treating kidney stones has been greatly improved by the development of various minimally invasive techniques. Currently, stone treatment is relatively mature. However, most current treatment methods are limited to stones and cannot effectively reduce their incidence and recurrence. Therefore, preventing disease occurrence, development, and recurrence after treatment, has become an urgent issue. The etiology and pathogenesis of stone formation are key factors in resolving this issue. More than 80% of kidney stones are calcium oxalate stones. Several studies have studied the formation mechanism of stones from the metabolism of urinary calcium, but there are few studies on oxalate, which plays an equally important role in stone formation. Oxalate and calcium play equally important roles in calcium oxalate stones, whereas the metabolism and excretion disorders of oxalate play a crucial role in their occurrence. Therefore, starting from the relationship between renal calculi and oxalate metabolism, this work reviews the occurrence of renal calculi, oxalate absorption, metabolism, and excretion mechanisms, focusing on the key role of SLC26A6 in oxalate excretion and the regulatory mechanism of SLC26A6 in oxalate transport. This review provides some new clues for the mechanism of kidney stones from the perspective of oxalate to improve the understanding of the role of oxalate in the formation of kidney stones and to provide suggestions for reducing the incidence and recurrence rate of kidney stones.

## 1. Introduction

Kidney stones are one of the most prevalent diseases in urology worldwide, and their prevalence and incidence have gradually increased over the past few decades. A recent national cross-sectional survey revealed that the prevalence of kidney stones is 6.4%, with a prevalence of 6.5% among men and 5.1% among women. Approximately 1 in 17 adults have kidney stone disease (1). Epidemiological data in the United States also revealed a high prevalence of kidney stones (2); the overall prevalence is rising annually (3), and stones easily relapse easily after treatment. The recurrence rate of kidney stones is estimated to be as high as 50% (3), and O Kamihira et al. (4) found that the recurrence rates of patients with kidney stones after 1, 3, and 5 years were 6.7, 28.0, and 41.8%, respectively. Solborg E Ingvarsdottir et al. (5) found that the recurrence rates of kidney stones in children after 5, 10, 15, and 20 years were 26, 35, 41, and 46%, respectively. The formation of kidney stones is also associated with a higher risk of diseases such as hypertension (6), chronic kidney disease, and end-stage renal disease (7). Therefore, treating stones and stone-related diseases imposes a substantial burden on people’s health annually.

The basic steps in kidney stone formation are urine supersaturation, nucleation, crystallization, growth, and aggregation (8). Regardless of the mechanism of kidney stone formation, the chemical processes of nucleation and crystallization are essential for the formation and development of kidney stones. Randall’s plaque theory is widely accepted for forming kidney stones, and Randall’s plaque is a prerequisite for forming calcium oxalate kidney stones. The majority (approximately 75%) of calcium oxalate stones are attached to Randall’s plaque (9), which begins with the deposition of calcium phosphate crystals within the renal interstitium. Calcium phosphate crystals are deposited on the basement membrane of the Henle loop and continuously mineralized to form subepithelial plaques. As the plaque gradually deposits, it penetrates the epithelium and is exposed in the urine of the renal papilla. Urinary metastable calcium oxalate binds to these plaques to form stones (10, 11).

## 2. Oxalate and kidney stones

More than 80% of kidney stones are calcium oxalate stones (10). The primary risk factors for calcium oxalate stones are hypercalciuria and hyperoxaluria. Supersaturation of urinary calcium oxalate is the driving force for forming calcium oxalate stones. Studies have investigated the formation mechanism of stones from urinary calcium metabolism, whereas relatively few studies have been conducted on oxalate, another important raw material for forming kidney stones. Oxalate plays an equal or even crucial role in calcium oxalate stone formation. Studies have reported that oxalate plays a greater role than calcium in forming calcium oxalate stones (12). In the balance between calcium ions, oxalate ions, and oxalate in urine, the oxalate concentration in urine is crucial. Small changes in urinary oxalate concentration can influence the formation of calcium oxalate crystals (12–14). Hyperoxaluria not only promotes the deposition of calcium oxalate crystals but also damages renal tubular epithelial cells through oxidative stress, thereby facilitating crystal adhesion. The subsequent immune inflammatory response after injury also promotes the formation of Randall plaques (15). Some studies have demonstrated that hyperoxaluria promotes transforming renal tubular epithelial cells into osteoblast phenotypes and forming Randall plaques and stones (16). In conclusion, stone formation results from a series of events, and hyperoxaluria, the initiating and promoting factor of calcium oxalate stone formation, is present throughout the process. Therefore, exploring the cause of hyperoxaluria is crucial for preventing oxalate stones. This review begins with the various causes of high hyperoxaluria, then discusses the source and removal of oxalate, as well as the factors affecting the source and removal of oxalate.

## 3. Evidence acquisition and synthesis

This review assessed the latest research articles published in PubMed between 2013 and 2023 and used PubMed to systematically review the original articles. We used the following search strategies: (oxalate) AND (SLC26A6), OR (oxalate) AND (excretion), OR (oxalate) AND (absorption), OR (oxalate) AND (oxalobacter), OR (primary hyperoxaluria) AND (kidney stone), OR (oxalate) AND (hyperoxaluria) AND (kidney stone), 1,075 manuscripts were retrieved from the literature. We reviewed these manuscripts and prioritized studies that fit our subject and those that are scientifically detailed and well-reported to help us understand them. Finally, 140 manuscripts were selected for our study.

## 4. Source of oxalate

**Figure 1:**
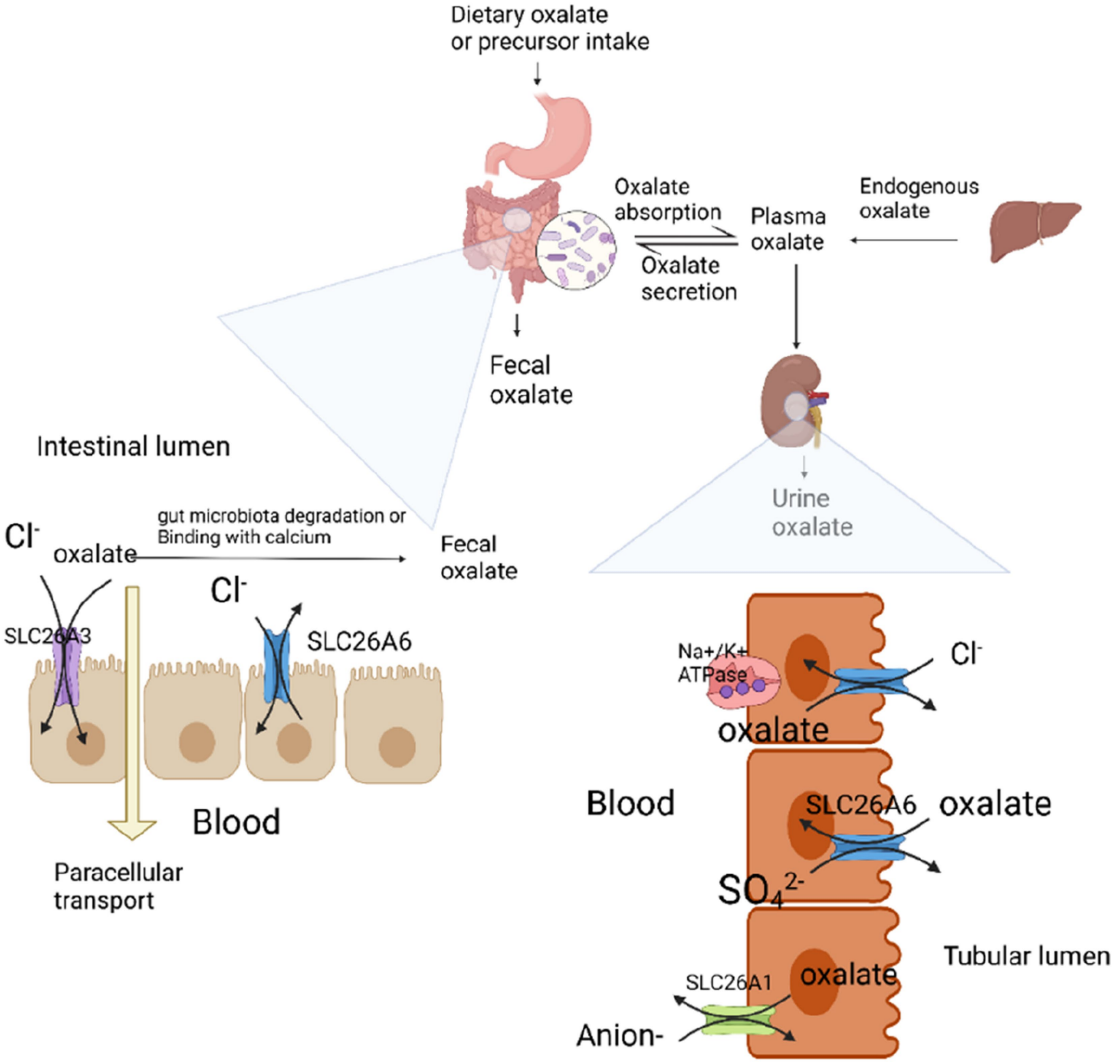
Absorption mechanism of oxalate in the intestine and kidney. Plasma oxalate is derived from endogenous oxalate produced by the liver and oxalate and its precursors absorbed by the intestine. In the intestine, dietary-derived oxalate promotes oxalate absorption through SLC26A3-mediated and paracellular absorption. SLC26A6-mediated oxalate secretion limits the net absorption of oxalate. Intestinal oxalate-degrading bacteria decompose oxalic acid to reduce the absorption of oxalic acid; calcium ions combine with oxalate to form insoluble calcium oxalate, which is not absorbed by the intestine and is excreted with feces, thus limiting the absorption of oxalate. Fatty acids reduce calcium combined with oxalate by binding to calcium, thereby promoting oxalate absorption. Oxalate excretion is assisted by the kidneys and intestine. In the kidney, oxalate is mainly excreted through glomerular filtration and can be assisted by SLC26A6-mediated oxalate secretion. Oxalate is in dynamic equilibrium under the influence of these factors. When some of these factors change, plasma oxalate concentration and urinary oxalate excretion increase, thereby promoting the formation of kidney stones.

Oxalate sources can be classified as exogenous or endogenous (Figure 1). Dietary oxalate from exogenous sources is absorbed by the intestine. Approximately 20–40% of blood oxalate is derived from the diet. Endogenous sources include liver, red blood cells, and ascorbic acid. Oxalate is produced by metabolism (17). Any means of producing excessive oxalate can cause high oxalate urine disease and promote oxalate stone formation. Primary hyperoxaluria (PH) is caused by excessive secretion of liver oxalate because of hereditary glyoxylate metabolic disorders. Secondary hyperoxaluria is caused by dietary intake of oxalate and oxalate precursors or factors that increase the net absorption of oxalate in the gastrointestinal tract (i.e., low calcium diet, poor fat absorption, and intestinal flora) (18).

### 4.1. Endogenous sources of oxalate and primary hyperoxaluria

**Figure 2:**
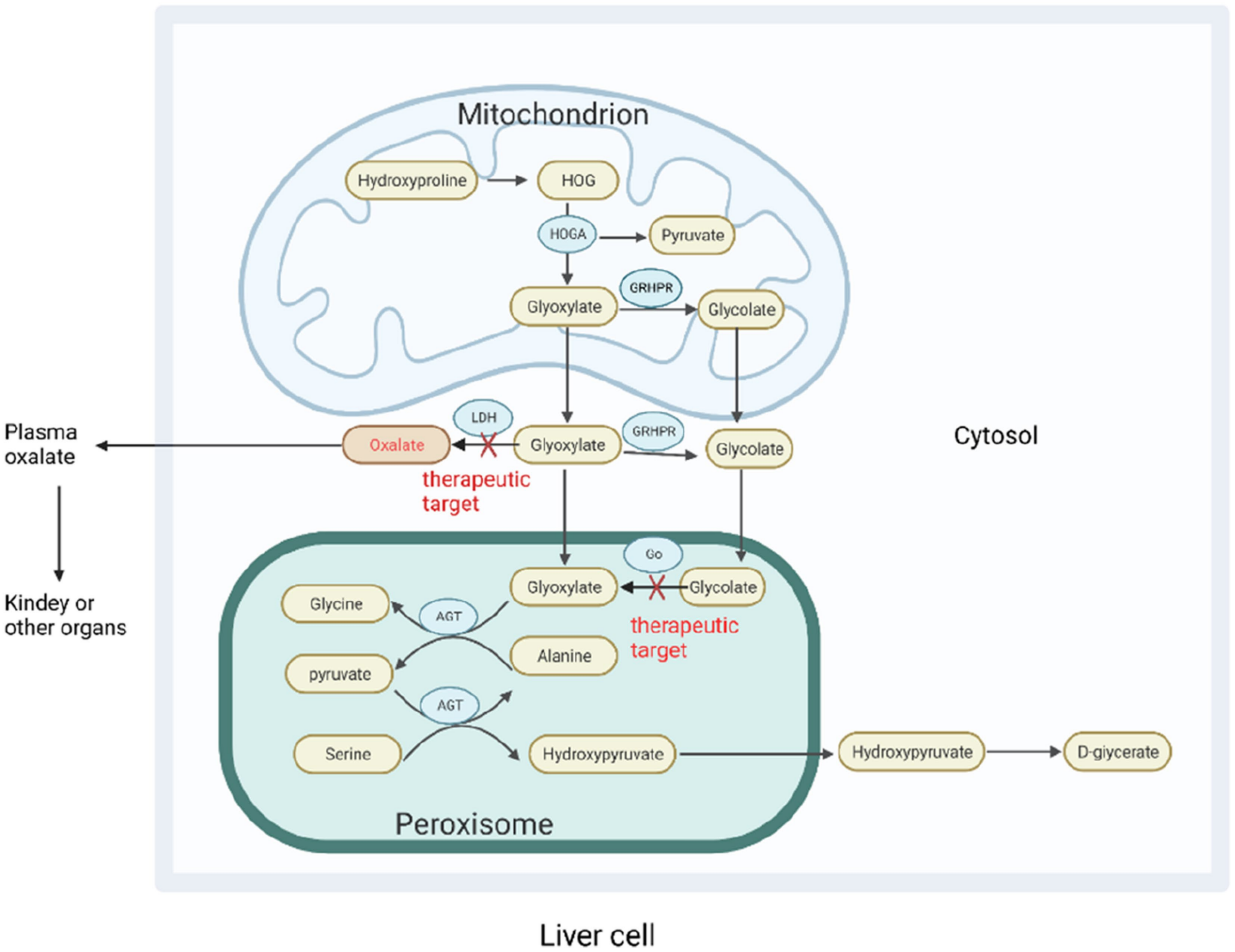
Oxalate metabolism in the liver. In hepatocytes, various oxalate precursors are metabolized into the direct precursor glyoxylate, which is then converted to oxalate by LDH. Glyoxylate can be catalyzed by various enzymes to reduce oxalate production. AGT catalyzes the conversion of glyoxylate and alanine to pyruvate and glycine, and glyoxylate can be used by cytosolic glyoxylate reductase-hydroxypyruvate reductase (GRHPR) to produce glycolate. Glycolate can also be metabolized by glycolate oxidase (GO) to produce glyoxylate. HOGA catalyzes the conversion of 4-hydroxy-2- oxoglutarate (HOG) produced by hydroxyproline in mitochondria to produce pyruvate and glyoxylic acid. In the absence of AGT and GRHPR, glyoxylate accumulates in the liver to produce excessive oxalate, resulting in primary hyperoxaluria types 1 and 2. The mechanism by which the absence of HOGA causes primary hyperoxaluria type 3 is unknown. One theory suggests that 4-hydroxy-2-oxoglutarate is broken down into oxalates without HOGA, whereas another suggests that it inhibits GRHPR activity in mitochondria. Go, and LDH are emerging targets for RNA interference-based treatment of primary hyperoxaluria. AGT, Alanine-glyoxylate aminotransferase; GRHPR, Glyoxylate reductase–hydroxypyruvate reductase; HOGA, 4-Hydroxy-2-oxoglutarate aldolase; HOG, 4-Hydroxy-2-oxoglutarate; Go, Glycolate oxidase; LDH, Lactate dehydrogenase.

Approximately 60–80% of plasma oxalate is derived from endogenous oxalate produced by liver metabolism (19). The oxalate precursor in the body is metabolized by the liver to produce glyoxylate, which is then converted to oxalate by lactate dehydrogenase (LDH). Alanine-glyoxylate aminotransferase (AGT) is a peroxisome enzyme in the human body that converts glyoxylate to glycine and plays a central role in reducing endogenous oxalate production. Glyoxylate reductase–hydroxypyruvate reductase (GRHPR) also metabolizes glyoxylates and helps limit the production of oxalates; 4-hydroxy-2-oxoglutarate aldolase (HOGA) is a key enzyme in the metabolism of hydroxyproline (20) (Figure 2). In the absence of these enzymes, glyoxylate is metabolized in the liver to produce an excess of endogenous oxalate, resulting in increased urinary oxalate excretion. Oxalate accumulates in various organs, including the kidney, in a rare autosomal recessive genetic disease known as primary hyperoxaluria (18). Primary hyperoxaluria is divided into types 1, 2, and 3, which are caused by a genetic deficiency of AGT, GRHPR, and HOGA, respectively. Primary hyperoxaluria type 1 is the most common and severe. Primary hyperoxaluria results in the accumulation of a large amount of oxalate to accumulate in the kidney, resulting in the formation of kidney stones and impairment of renal function, eventually leading to end-stage renal disease.

### 4.2. Exogenous sources of oxalate

Approximately 20–40% of the oxalate in the plasma is derived from the diet absorbed through the intestine. Although exogenous oxalate accounts for a relatively small proportion of total plasma oxalate, it is susceptible to various factors and, thus, is highly variable and can be easily controlled by humans. Controlling exogenous oxalate is a feasible method for preventing stone formation. After feeding hyperoxaluria mice with an oxalate-free diet, hyperoxaluria substantially improved, confirming that most urinary oxalates were derived from the intestines (19). Therefore, we must understand the absorption mechanism of oxalate in the intestine and the factors that affect its absorption.

#### 4.2.1. Absorption mechanism of oxalate in the intestine

Whether oxalate absorption in the intestine is paracellular or transcellular or has not been determined. Knauf F et al. (21) found that the absorption flux of oxalate in the mouse intestine is similar to that of mannitol (mannitol is a marker of the paracellular pathway), is insensitive to the anion transport inhibitor DIDS, and is unsaturated. Therefore, oxalate is passively absorbed in the intestine through the paracellular pathway, and the net absorption of oxalate depends on the relative balance between the absorption of the paracellular pathway and the secretion of oxalate dependent on intestinal SLC26A6. However, Robert W. Freel et al. (22) compared the one-way and net fluxes of oxalate ions in the intestine of wild-type (WT) and DRA knockout (KO) mice and found that in WT mice, all intestinal segments exhibited varying degrees of oxalate net absorption. However, in KO mice, oxalate absorption flux was significantly decreased, whereas secretion flux did not change significantly. All segments exhibited oxalate net secretions. The daily urinary oxalate excretion of KO mice was 66% lower than that of WT mice, indicating that the net secretion of oxalate in KO mice was due to a decrease in oxalate absorption flux. Accordingly, DRA (SLC26A3) protein mediates intestinal absorption of oxalate across cellular pathways. Robert W. Freel (21) explained that these two distinct results may be due to technical differences and that shunt permeability may be exaggerated in his study. If oxalate uptake is a paracellular passive transport, the relationship between oxalate uptake flux and conductance (GT) should be linear, with an intercept of zero. Whittamore et al. (23) found that the relationship between oxalate absorption flux and GT in the distal colon of WT mice was indeed linear but that its intercept did not pass zero and that the oxalate absorption flux when GT was zero was very similar to that mediated by SLC26A3 (22). These findings indicate that the oxalate absorption flux at GT = 0 may be mediated by SLC26A3 and that oxalate absorption may occur through paracellular and transcellular pathways.

#### 4.2.2. Factors affecting intestinal absorption of oxalate

The intestinal absorption of oxalate is influenced by many factors, including the role of intestinal microbes, the form of oxalate, and intestinal fat absorption. A low-calcium diet is considered a risk factor for oxalate stones because intestinal calcium can be combined with oxalate to form insoluble calcium oxalate, which is difficult to be absorbed and discharged in feces. When the intestine lacks calcium, more oxalate becomes free, and these free oxalates are more easily absorbed, leading to hyperoxalemia (24–26). A recent report described a case of hyperoxaluria induced by a calcium-free diet. After calcium was added to the diet, the concentration of urine oxalate decreased substantially. This report indicates the importance of calcium intake in preventing calcium oxalate kidney stones (27). Moreover, when fat absorption is poor or a high-fat diet is consumed, excessive free fatty acids can be combined with dietary calcium, thereby increasing the free oxalate content and absorption (28, 29). Fatty acids also increase the permeability of local mucosa, thereby promoting oxalate absorption (30). The incidence of hyperoxaluria and calculus increased substantially after Roux-en-Y gastric bypass and malabsorption bariatric surgery. One of the primary causes may be inadequate fat absorption (31–34). Hyperoxaluria is often observed in digestive diseases associated with poor fat absorption, such as Crohn’s disease (35, 36), biliary tract disease (30), pancreatic disease (37), and short bowel syndrome (38). In patients with fat malabsorption, supplementation with fat-soluble vitamins negatively correlates with urinary oxalate excretion (39).

#### 4.2.3. Intestinal microbes and oxalate metabolism

Because hyperoxaluria is largely intestinal in origin, it is possible to prevent calcium oxalate stones by increasing the degradation of intestinal oxalate, thereby reducing intestinal oxalate absorption. However, the human body lacks an oxalate degradation pathway, and the required oxalate-degrading enzymes must be obtained exogenously. Oxate-degrading bacteria with the oxalate-degrading genes *frc* and *oxc* are found in the human intestine (40). These oxalate-degrading bacteria can use oxalate as an energy source to reduce the content of absorbable oxalate in the intestine, thereby reducing urinary oxalate excretion (41) (Table 1). Oxalate-degrading bacteria include “specialist oxalotroph” such as *Oxalobacter formigenes* and “generalist oxalotrophs” such as *Bifidobacterium animalis*, *Lactobacillus acidophilus*, and *Lactobacillus gasseri* (54). Benjamin K Canales et al. (51) colonized intestinal *O. formigenes* in model rats with hyperoxaluria after Roux-en-Y gastric bypass and found that urinary oxalate excretion decreased by 74% compared with the uncolonized group. Bernd Hoppe et al. (59) administered *O. formigenes* oral preparations and placebo for 43 patients with primary hyperoxaluria. The urinary oxalate content of patients who consumed Oxalobacter oral preparations decreased by 19% compared to before treatment within 24 h. Furthermore, Several studies have examined the effect of intestinal colonization of oxalate-degrading bacteria on hyperoxaluria and have demonstrated that intestinal colonization with oxalate-degrading bacteria has a certain effect on the prevention and treatment of hyperoxaluria (45, 58, 60–64). Therefore, colonization of the intestine with oxalate-degrading bacteria may reduce intestinal oxalate absorption, plasma oxalate concentration, and urinary oxalate excretion, which is a potential strategy for preventing oxalate stones.

**Table 1:** Treatment of hyperoxaluria and kidney stones with intestinal flora.

Research subject	Method	Result	Conclusion	Reference
PH patients	Subjects were randomized to receive Oxabact(a lyophilized *O. formigenes* formulation) or placebo	Plasma oxalate levels decreased in subjects treated with *O. formigenes*	*O. formigenes* can reduce plasma oxalate and help prevent kidney stones	(42)
PH patients	Subjects were randomized to receive O.formigenes or placebo	Compared with placebo, there was no significant difference in urinary oxalate excretion and plasma oxalate concentration after O.formigenes treatment.	O.formigenes treatment did not significantly reduce urinary oxalate excretion or plasma oxalate concentration within 8 weeks of treatment	(43)
PH patients	Subjects were randomized to receive O.formigenes or placebo	Compared with placebo, there was no significant difference in urinary oxalate excretion and plasma oxalate concentration after O.formigenes treatment.	O.formigenes treatment did not reduce urinary oxalate during 24 weeks of treatment in PH patients	(44)
PH patients	Oral *O. formigenes* preparations	All subjects had reduced compared with baseline	O.formigenes is beneficial to PH1 subjects, which can significantly reduce plasma oxalate concentration, improve or stabilize cardiac function and clinical status	(45)
PH infant patients	Hemodialysis combined with oral O.formigenes	Compared with before treatment, plasma oxalate decreased significantly.	*O. formigenes* preparations contribute to the treatment of PH.	(46)
PH infant patients	Hemodialysis combined with O.formigenes	Plasma oxalate concentration decreased	Hemodialysis combined with *O. formigenes* treatment can reduce plasma oxalate, stabilize systemic oxalosis, and improve the clinical course.	(47)
Urolithiasis patients and healthy controls	Detection of fecal oxalate-degrading bacteria abundance	The relative abundance of fecal *O. formigenes* in the kidney stone group was lower than in the control group.	*O. formigenes* is important in inhibiting the formation of kidney stones and hyperoxaluria.	(48)
Urolithiasis patients and healthy controls	Fecal *O. formigenes* abundance and urinary oxalate levels were detected.	Urinary oxalate excretion was less in patients with intestinal colonization than in patients without intestinal colonization	Lack of *O. formigenes* can lead to a significant increase in the risk of hyperoxaluria and lead to recurrent calcium oxalate stone attacks.	(49)
Urolithiasis patients and healthy controls	Detection of fecal O.formigenes abundance	Compared with the control group, the colonization of O.formigenes in patients with renal calculi decreased.	The lack of O.formigenes colonization may be an important risk factor for stone formation and recurrence	(50)
Urolithiasis patients with hyperoxaluria	Received daily a mixture containing freeze-dried lactic acid bacteria.	Compared with before treatment, Urinary oxalate excretion was significantly reduced.	Oral freeze-dried lactic acid bacteria is a method for treating and preventing kidney stones.	(41)
Roux-en-Y gastric bypass model rats with hyperoxaluria	Intragastric administration of *O. formigenes*	*O. formigenes* colonization reduced urinary oxalate excretion in RYGB rats.	*O. formigenes* colonization degraded intestinal oxalate and promoted intestinal oxalate elimination	(51)
Primary hyperoxaluria type 1 model mouse	Rectal administration of *O. formigenes*	Urinary oxalate excretion decreased, and distal colonic oxalate secretion increased.	*O. formigenes*-derived bioactive factors stimulate oxalate transport in intestinal cells, which has therapeutic potential.	(52)
Rat model of primary hyperoxaluria	Kidney stone model rats oral *O. formigenes* feed	O.formigenes treatment partially prevented ethylene glycol-induced increases in plasma and urine oxalate and completely prevented renal calcinosis.	*O. formigenes* has beneficial effects on ethylene glycol-induced hyperoxalemia and renal calcinosis, thus supporting the beneficial role of *O. formigenes* in primary hyperoxaluria.	(53)
Primary hyperoxaluria rats with slc26a6 knockout or slc26a3 knockout	Intestinal colonization of *O. formigenes*	*O. formigenes* can induce intestinal oxalate excretion in the absence of either apical oxalate transporter and reduce urinary oxalate excretion.	*O. formigenes* oxalate-induced intestinal oxalate secretion in mice does not require the presence of apical oxalate transporter SLC26A6 or SLC26A3, and other unidentified oxalate transporters are involved in mediating oxalate transport.	(54)
mice	A human *O. formigenes* strain (HC-1) colonized mice	HC-1 promoted strong net secretion of oxalate in the terminal ileum, cecum and colon of mice and reduced oxalate excretion in the kidney.	HC-1 has great potential as a probiotic treatment for hyperoxaluria.	(55)
a mouse model of Primary Hyperoxaluria	Intestinal colonization of Bifidobacterium in PH model rats	Urinary oxalate excretion was significantly reduced.	Bifidobacterium has the potential to prevent and treat hyperoxaluria.	(56)
Hyperoxaluria rat model	Intragastric administration of *O. formigenes*	Urinary oxalate excretion decreased and was proportional to the dose of *O. formigenes*.	Probiotic treatment of rats with hyperoxaluria can significantly and rapidly reduce urinary oxalate levels.	(57)
Rat model of primary hyperoxaluria	Intestinal colonization of *O. formigenes*	Intestinal colonization of *O. formigenes* significantly reduced plasma and urinary oxalate excretion.	Plasma and urinary oxalate returned to normal after colonization of oxalate in hyperoxaluria rats.	(58)

These oxalate-degrading bacteria affect the absorption of oxalate not only by decomposing oxalate but also by specific metabolites. Short-chain fatty acids (SCFAs), the main product of intestinal microbial fermentation, can regulate oxalate absorption, thereby reducing urinary oxalate excretion and calcium oxalate stone formation. Yu Liu et al. administered SCFAs to calcium oxalate model rats and observed a decrease in kidney crystals (65). Comparing patients with calcium oxalate kidney stones and the control group, the control group had more bacteria with SCFAs metabolites in the intestine (48, 65). SCFAs can increase the expression of intestinal SLC26A6 and reduce the expression of intestinal SLC26A3, thereby affecting the absorption and excretion of oxalate and reducing the formation of urinary oxalate and calcium oxalate kidney stones (66). Furthermore, *O. formigenes* can not only degrade oxalate to reduce oxalate absorption but also increase the intestinal secretion of oxalate (52, 54). However, healthy subjects ingesting lactic acid bacteria preparations after consuming a high oxalate diet demonstrated no reduction in urinary oxalate excretion or plasma oxalate concentration (67). A phase I/II/III randomized trial on the efficacy and safety of colonization of *O. formigenes* in the treatment of primary hyperoxaluria revealed no significant reduction in urinary oxalate excretion and plasma oxalate concentration after treatment with lithogenic oxalate preparation (43, 44). Therefore, the mechanism of oxalate-degrading bacteria degrading intestinal oxalate requires further investigation.

Although considerable research on the intestinal metabolism of oxalates has focused on oxalate-degrading bacteria, recent studies—with the advancement of sequencing technology have demonstrated that the microbiota plays a broader role in oxalate metabolism and the prevention of urinary stones (68). Studies have reported no significant difference in the number of intestinal oxalate bacilli between patients with hyperoxaluric kidney stones and normal controls; however, there is a reduction in intestinal microbial diversity, a significant reduction in the number of bacteria associated with oxalate degradation genes, and a change in the composition of intestinal microbes (48, 69–71). Animal models and human experiments have demonstrated that the risk of urinary calculi is significantly increased after taking antibiotics and reducing the bacterial load of the intestine (65, 66, 72). Intestinal microbial depletion may promote urinary oxalate excretion and calcium oxalate kidney stone formation. Consequently, gastrointestinal oxalate homeostasis is not only attributed to *O. formigenes* but may also involve the combined action of many bacterial species, which may help explain why previous probiotic intervention studies failed to reduce the risk of hyperoxaluria (43, 44, 63, 73, 74).

In summary, intestinal *O. formigenes* and other probiotics have a certain effect on oxalate degradation, but their individual effects may be limited. The role of probiotics depends largely on the diversity and integration of intestinal microorganisms. Numerous species of intestinal microorganisms can degrade oxalate. They can interact with each other to form an intestinal microbial network, and oxalate metabolism is not limited to a few species (68, 69, 73, 75). However, the roles of *O. formigenes* cannot be ignored. *O. formigenes* is the center of the microbial network involved in oxalate degradation and prevention of hyperoxaluria (73). Therefore, successful development of bacterial therapies to inhibit urinary stones requires a strategy that targets bacterial diversity rather than a few specific species.

## 5. Oxalate excretion

In the absence of enzymes for oxalate metabolism in the body, oxalate, as a metabolic end product, must be excreted from the body (17). Oxalate was eliminated through renal and intestinal excretion pathways (Figure 1). The kidneys excrete most of the oxalate in the body, whereas the intestines excrete only a small amount. Hyperoxaluria can be caused by metabolic defects in the kidney and intestine. Disorders of intestinal oxalate secretion disorder or enhanced renal excretion may cause high oxalate urine, leading to kidney stones. The anion transporter SLC26A6 is essential in oxalate excretion in the kidneys and intestines.

### 5.1. Structure and physiological function of SLC26A6

Solute-linked carrier 26 gene family 6 protein (SLC26A6) is a multifunctional anion exchanger. It has the most extensive transport function in the SLC26A family and can transport a variety of anions (76). SLC26A6 is a transmembrane secondary transporter comprising 759 amino acids. The membrane insertion domain consists of 14 variable-length α-helixes, which are organized into two structurally related regions. Each region consists of seven transmembrane segments, thereby forming an interwoven structure consisting of two inverted repeats (77). A STAS domain is present at the C-terminus, which is related to intracellular transport and protein–protein interactions, and its deletion damages the substrate transport of the membrane domain (78, 79). Furthermore, the C-terminus of SLC26A6 contains a common PDZ interaction motif identical to that of the cystic fibrosis transmembrane conductance regulator (CFTR). The PDZ domain provides a site for protein–protein interactions, which are essential in assembling multiprotein complexes (80). SLC26A6 is highly expressed in the human intestine, kidney, other tissues, and the mammalian heart, reproductive system, esophagus, stomach, and other tissues. SLC26A6 is a multifunctional anion transporter and mediates the transport of anions such as Cl^−^/HCO_3_^−^, Cl^−^/formic acid, Cl^−^/oxalate, Cl^−^/nitrate, SO₄^2−^/oxalate, and Cl^−^/OH^-^ (81, 82). It plays an essential role in ion balance and acid–base homeostasis, and its dysfunction is closely related to various diseases in various systems. SLC26A6 exhibits complete transport activity and can perform its normal function only after glycosylation. *In vitro*, studies have demonstrated that SLC26A6 is highly N-glycosylated, and N-glycosylation has an important effect on the folding, transport, and function of various membrane proteins (83). R. Brent Thomson (84) found that SLC26A6 is glycosylated and tissue-specific when endogenously expressed in mice and humans. Glycosylation of human SLC26A6 on promoters 167 and 172 is essential for oxalate transport. When SLC26A6 is deglycosylated by glycosidase, oxalate transport activity is significantly decreased, indicating that the oxalate transport function of SLC26A6 is heavily dependent on glycosylation. Glycosylation plays a key regulatory role in the transport function of SLC26A6. SLC26A6, which is expressed in the kidney and intestine, mediates the secretion and absorption of oxalate and is the most important channel protein involved in oxalate transport (85). Abnormal expression and function of SLC26A6 in the intestine and kidney are closely related to hyperoxalemia, hyperoxaluria, and renal calcium oxalate stones.

### 5.2. Oxalate excretion in the kidney and stone formation

The kidney is the primary pathway for oxalate excretion. Oxalate is mainly excreted through glomerular filtration (86), but renal tubular secretion can also assist in oxalate excretion (87, 88). For normal people, oxalate is freely filtered in the glomeruli and is not easily regulated, and the oxalate secretion of renal tubules must be mediated by SLC26A6 (89). Therefore, differences in SLC26A6 expression can cause differences in oxalate secretion, and the regulation of SLC26A6 expression may affect oxalate secretion in proximal tubules. SLC26A6 is located on the lumen side of the proximal tubule and transports oxalate from the cell to urine through Cl−/oxalate exchange, that is oxalate secretion. It can also transport oxalate from urine to the cell through SO₄^2−^ /oxalate exchange, which means oxalate reabsorption (17). Perfusion studies have demonstrated that S1 and S2 segments of the proximal tubule of rats absorb oxalate, whereas S3 segment secretes oxalate (87, 90). Abnormal expression of SLC26A6 in the kidney may cause oxalate excretion disorders, resulting in high oxalate urine and the formation of oxalate stones. Jiang et al. reported a significant increase in the expression of SLC26A6 in the kidneys of stone patients compared to that in controls (91). By targeting up-regulation and down-regulation of SLC26A6 expression in the kidneys of mouse stone models, the results demonstrated that urinary oxalate concentration and stone formation rate significantly increased in SLC26A6 up-regulated mice (91). In oxalate-treated NRK-52E cells, after up-regulation of SLC26A6 expression, cell viability decreased more significantly, apoptosis rate increased significantly, and ROS and SOD production increased (92). *In vivo* and *in vitro* experiments concluded that the increase in SLC26A6 expression in the kidney can cause an increase in the secretion of oxalate in renal tubular epithelial cells, increasing the concentration of oxalate in urine. High oxalate concentrations in urine can damage renal tubular epithelial cells through oxidative stress, and damaged renal tubular epithelial cells are more likely to cause crystal adhesion and aggregation. The two factors of Oxalate oversaturation in urine and damage to renal tubules reinforce each other, eventually leading to the formation of oxalate stones. Studies have found that glycine can reduce urinary oxalate and significantly reduce calcium oxalate crystal deposition in rat kidneys induced by ethylene glycol. Mechanistic studies have demonstrated that glycine reduces urinary oxalate excretion by down-regulating the expression of SLC26A6 in the kidney (93). Therefore, increased expression of SLC26A6 in the kidney is one of the causes of oxalate stones, and down-regulation of SLC26A6 is a potential strategy for preventing oxalate stones.

### 5.3. Oxalate excretion in the intestine and stone formation

The intestinal tract, as an auxiliary pathway of renal excretion of oxalate, is of great significance in limiting the net absorption of oxalate by the intestinal tract, reducing the excretion of oxalate in the kidney, thereby reducing the concentration of oxalate in the urine and preventing the occurrence of oxalate stones. The secretion of oxalate in the intestine needs to be mediated by SLC26A6 (94, 95). Zhirong Jiang et al. found a significant incidence of hyperoxalemia, hyperoxaluria, and oxalate stones in SLC26A6 KO mice (94). The mechanism may be that in SLC26A6 gene KO mice, due to the deficiency of SLC26A6-mediated oxalate secretion in the intestine, the net absorption of oxalate in the intestine increases, the concentration of oxalate in the plasma increases, and more oxalate is excreted from the urine, causing hyperoxaluria, which leads to the occurrence of oxalate stones. Robert W. Freel et al. also found a similar conclusion. In WT mice, the ileum had a net secretion of oxalate, whereas, in SLC26A6 KO mice, the ileum was converted to a net absorption of oxalate. The urinary oxalate excretion of SLC26A6 KO mice was four times that of WT mice. By administering the inhibitor DIDS of the mouse anion transporter SLC26A6, the ileum of WT mice changed from net secretion of oxalate to net absorption (95). Subsequent studies have reported similar findings that SLC26A6 gene defects lead to urolithiasis (96, 97). In a mouse model of chronic kidney disease, SLC26A6-mediated intestinal oxalate secretion is essential to reduce the body burden of oxalate (98). A net secretion of oxalate exists in the distal colon of mice, but it does not require the involvement of SLC26A6 and may have other unknown transporters (99). SLC26A6 overexpression in the kidney increases urinary oxalate excretion and promotes stone formation, whereas overexpression of SLC26A6 in the intestine increases intestinal oxalate secretion, reduces urinary oxalate excretion, and plays a protective role in stone formation. Therefore, SLC26A6 is expected to be a target for stone treatment and prevention. The formation of kidney stones can be prevented by up-regulating or down-regulating SLC26A6 expression.

## 6. SLC26A6 gene mutation and oxalate stones

SLC26A6 is essential for limiting the net absorption of oxalates by the gut and preventing kidney stones. Therefore, functional defects and mutations in human SLC26A6 may cause hyperoxaluria and kidney stones. Five studies have reported the effect of SLC26A6 variants on human kidney stones (100–104). The first study reported the detection of six missense mutations in the SLC26A6 gene in the case and control groups. Among them, C.616G > A (p.Val206 Met) is the most common mutation (11%), but the mutation does not affect the population plasma or urine oxalate (100). The second study reported the relationship between SLC26A6 gene 206 M polymorphism and the risk of renal calculi in PHPT patients. The results also demonstrated that SLC26A6 gene 206 M polymorphism was not associated with renal calculi in PHPT patients (101). Non-homologous single nucleotide polymorphisms (nsSNPs) associated with kidney stones in the SLC26A6 gene were screened using Silico Screening by Xiuli Lu et al. (102), and nsSNP rs184187143 was identified as a more likely disease-related variation in SLC26A6 gene. In nsSNP, the risk of kidney stones increased by 6.1 times in C allele carriers compared with that of G allele carriers. Liana et al. (103) reported two new polymorphisms in the STAS domain of SLC26A6. This mutation impairs the regulation of NADC-1-mediated citrate transport by weakening the interaction between the STAS domain of SLC26A6 and NADC-1, causing hypocitraturia and occurrence of calcium oxalate kidney stones. Nicolas Cornière et al. (104) recently reported a rare heterozygous missense mutation (c.1519C > T/p.R507W) in SLC26A6 gene in a patient with calcium oxalate nephrolithiasis who had significant hyperoxaluria. R507W mutation was transfected into OKP cells, and the mutation resulted in decreased expression of SLC26A6 and significantly impaired oxalate transport activity. This result indicates that the p. R507W mutation affected the expression and transport activity of SLC26A6.

## 7. SLC26A6-mediated regulation of oxalate transport

**Figure 3:**
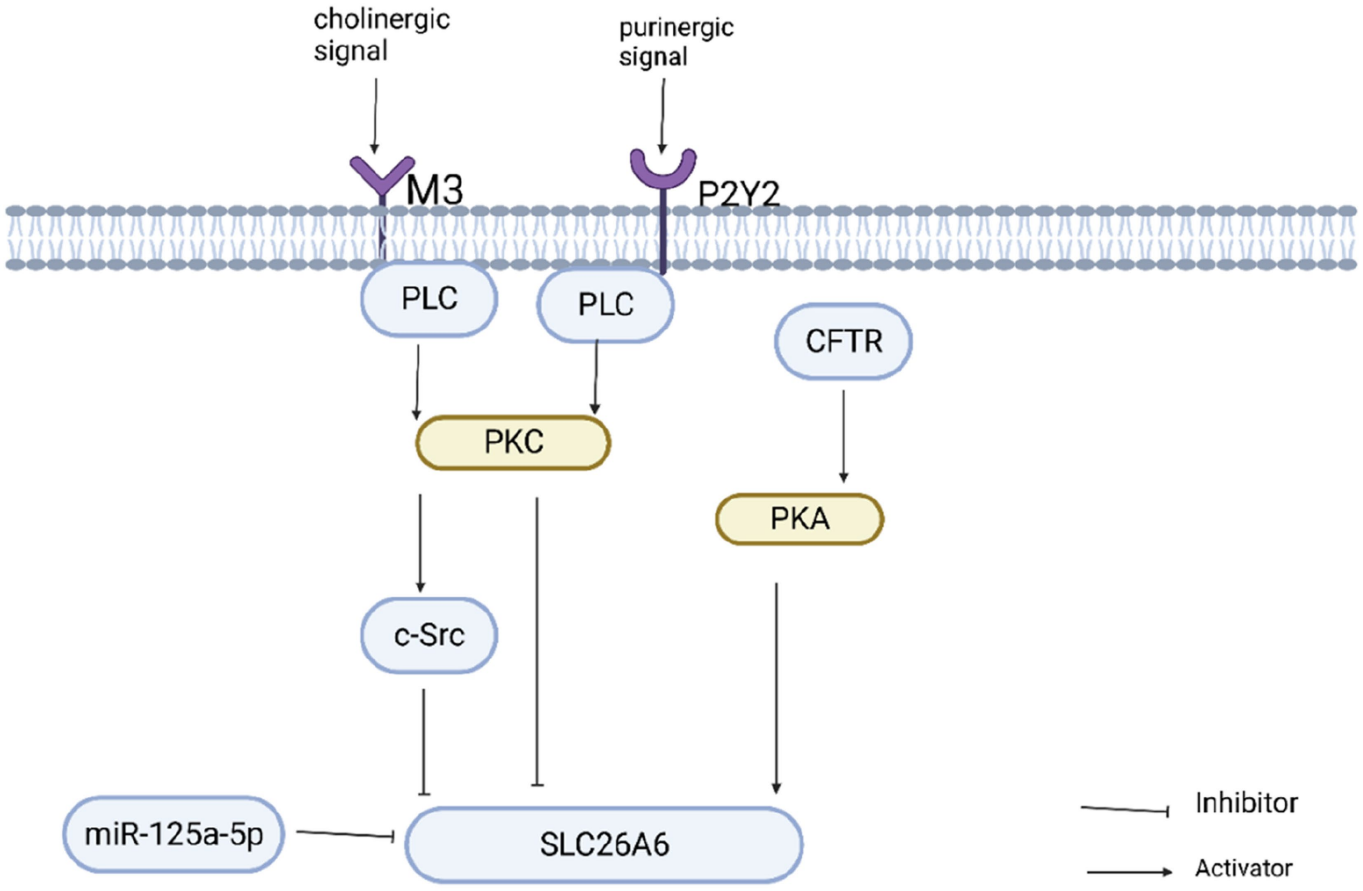
Regulation of slc26a6 oxalate transport activity.

SLC26A6-mediated oxalate secretion is essential in oxalate excretion in the kidney and intestine. Increased oxalate secretion in the kidney promotes stone formation, whereas increased oxalate secretion in the intestine inhibits stone formation. Therefore, elucidating the regulatory mechanism of SLC26A6 is essential for preventing calcium oxalate kidney stones. SLC26A6 is activated or inhibited through multiple signaling pathways. Through the study of these pathways, the expression and activity of SLC26A6 in the kidney and intestine can be selectively regulated, which is a potential strategy for preventing stones (Figure 3). Studies have demonstrated that cholinergic signals can inhibit the expression of SLC26A6 on the cell surface through signaling pathways including the M3 receptor, phospholipase C, PKC-β, and c-Src, thereby negatively regulating oxalate transport (105). Similarly, purinergic signaling can also inhibit oxalate transport by activating P2Y2 purinergic receptors, PLC, and PKC signaling pathways to reduce the surface expression of SLC26A6 (106). SLC26A6 can also be regulated by microRNAs, which are a group of small non-coding RNAs that regulate gene expression by binding to the 3′untranslated region (3′UTR) of target mRNAs. Arivarasu N (107) found that miR-125a-5p inhibited the expression of SLC26A6 by directly interacting with the 3′UTR of SLC26A6 mRNA and inhibited the transport of oxalate. SLC26A6 is activated by various regulatory factors. Cystic fibrosis protein (CFTR) is also closely related to the activation of SLC26A6. CFTR may promote the expression of SLC26A6 (79, 108). Felix Knauf et al. (108) found that the expression of SLC26A6 decreased in the duodenum of *CFTR* gene-deficient mice, intestinal oxalate secretion decreased significantly, and the deficient mice exhibited hyperoxalemia and hyperoxaluria. Estradiol can up-regulate CFTR on the surface of intestinal mucosal cells to increase the expression of SLC26A6 and regulate oxalate transport (109, 110). Therefore, CFTR may be an upstream protein of SLC26A6. Furthermore, SLC26A6 is regulated through the PKA activation signal. After activating PKA, the expression of intestinal SLC26A6 was significantly increased, and its transport activity was enhanced (52, 111). CFTR can activate PKA (79) and SLC26A6 through signaling pathways such as PKA, but the exact mechanism requires further study. These results suggest that the expression of SLC26A6 and SLC26A6-mediated oxalate transport is positively and negatively regulated by various factors. Manipulating these regulatory factors and selectively regulating the expression and activity of SLC26A6 is a potential therapeutic approach. Furthermore, slc26a6 can interact with the citrate transporter NADC-1 to regulate the reabsorption of urinary citrate, thereby inhibiting the formation of calcium oxalate stones (85, 112).

## 8. Treatment and prevention strategies for calcium oxalate stones

In conclusion, to prevent hyperoxaluria and oxalate stones, the source and destination of oxalate must be investigated. First, we should reduce the source of exogenous oxalate and the generation of endogenous oxalate. Therefore, the intake of foods rich in oxalate and oxalate precursors should be reduced. However, oxalate is widely present in many foods, and it is difficult to control the intake. Only foods with very high oxalate content can be avoided or restricted. Second, reducing the intestinal absorption of oxalate is a method to prevent hyperoxaluria and oxalate stones. Therefore, one must avoid low calcium, high-fat diet and treatment of diseases that cause poor fat absorption; SLC26A3 plays an important role in the intestinal absorption of oxalate, which can be considered to down-regulate SLC26A3 expression in the intestine. Furthermore, intestinal flora plays an essential role in degrading oxalate and limiting the intestinal absorption of oxalate, but oxalate degradation is more dependent on the integration of intestinal flora. Therefore, the intervention measures to limit the absorption of oxalate should consider the complexity of intestinal microflora, not just one or two bacteria. Finally, starting with the oxalate excretion pathway, more oxalate can be excreted from the intestine to reduce oxalate excretion in the kidney. Oxalate transporters play essential roles in oxalate absorption and excretion. Among them, the study of oxalate transporter SLC26A6 is more in-depth. The secretion of oxalate mediated by SLC26A6 in the intestine limits the net absorption of oxalate in the diet and reduces oxalate excretion in urine, whereas oxalate secretion mediated by SLC26A6 in the kidney increases the excretion of oxalate in urine. Therefore, it is possible to target the up-regulation of SLC26A6 expression in the intestine and its down-regulation in the kidney, thereby strengthening the inhibition of stone formation and weakening stone formation. However, SLC26A6, as a multifunctional anion exchanger, should also consider whether it affects the transport of other ions while regulating its expression, resulting in severe consequences.

For stones caused by primary hyperoxaluria, active treatment of the primary disease is required. Conventional treatment of primary hyperoxaluria includes high fluid intake, calcium oxalate crystallization inhibitors, and vitamin B6 (113). Liver transplantation is the only curative therapy in the past. Inhibition of liver-specific glycolate oxidase or reduction of liver lactate dehydrogenase is a promising strategy for primary hyperoxaluria treatment. Emerging therapies based on RNA interference have significantly influenced the treatment and prognosis of patients with primary hyperoxaluria. Lumasiran, a small interfering RNA (siRNA), inhibits oxalate synthesis by silencing genes encoding glycolate oxidase (114). Recent clinical phase III studies have demonstrated that Lumasiran treatment significantly reduced plasma oxalate concentration and urinary oxalate excretion. Most patients have normal or near-normal urinary oxalate levels after 6 months of treatment (115). Lumasiran was effective during the treatment of PH1. Urine oxalate remained low at 12 and 18 months, and no adverse reactions were observed (116, 117). Furthermore, Nedosiran, as an RNA interference agent, can effectively reduce urinary oxalate excretion by specifically inhibiting LDH (a key enzyme in liver oxalate synthesis) so that glyoxylate cannot be converted into oxalate (118, 119). For the first time, Michelle A Baum et al.conducted a phase 1 clinical trial in humans. The results displayed that compared with before treatment, 24-h urinary oxalate excretion in PH patients decreased by an average of 55% after Nedosiran treatment, and 33% of subjects returned to normal (120). After that, Michelle A Baum et al.conducted a randomized study on treating PH with Nedosiran. The results also depicted that compared with the placebo group, plasma oxalate and urinary oxalate in PH patients treated with Nedosiran were significantly reduced, and Nedosiran was generally safe and well tolerated by patients (121). Developing these new therapies to cure metabolic defects is expected to eliminate the need for liver transplantation, greatly improving the quality of life for primary hyperoxaluria patients.

## 9. Conclusion and outlook

In light of the high incidence and recurrence rate of kidney stones, urologists focus more on treating stones than on their prevention. Future research must focus on etiology and pathogenesis to effectively prevent kidney stones, as the importance of prevention cannot be overemphasized. Calcium oxalate stones, as a major type of kidney stone, can effectively reduce the formation of stones through targeted intervention in the source and excretion of oxalate. Intestinal oxalate-degrading bacteria play an essential role in limiting oxalate absorption; however, their function depends largely on the integration of intestinal flora. The future development of bacterial therapy to prevent kidney stones must consider the entire intestinal flora; SLC26A6 is closely related to the excretion of oxalate and is expected to be a target for preventing and treating kidney stones. Its expression in the intestine promotes the excretion of oxalate in the intestine, thereby reducing the excretion of urinary oxalate, which has a protective effect on kidney stones, whereas its expression in the kidney promotes the excretion of oxalate in the kidney, thereby increasing urinary oxalate and promoting kidney stones. Future research must confirm the feasibility of targeted up-regulation or down-regulation of SLC26A6 gene in preventing and treating oxalate stones. Our current understanding of the etiology and pathogenesis of stones remains limited, necessitating further research in the future to enhance our knowledge in this area.

## Author contributions

BQ conceived the manuscript. TC searched publications and draft the manuscript. JunZ and PL edited tables and figures. WL and QC collect data. LZ and JunrZ reviewed the manuscript and polished the grammar. All authors contributed to the article and approved the submitted version.

## Funding

This work was supported by the scientific research project of Jiangxi Provincial Department of Education (GJJ2201403), the key scientific research project of Gannan Medical University (ZD201909), and the research project of the First Affiliated Hospital of Gannan Medical University (YJYB202111).

## Conflict of interest

The authors declare that the research was conducted in the absence of any commercial or financial relationships that could be construed as a potential conflict of interest.

## Publisher’s note

All claims expressed in this article are solely those of the authors and do not necessarily represent those of their affiliated organizations, or those of the publisher, the editors and the reviewers. Any product that may be evaluated in this article, or claim that may be made by its manufacturer, is not guaranteed or endorsed by the publisher.
